# The Medical Organization of the Italian Army

**Published:** 1919

**Authors:** 


***The Medical Organization of the Italian Army. The Neuropsychiatric Service.*** By SANTE NACCARATI. *The Journal of the American Medical Association*, November 2, 1918.

The Italian Medical Service outside the zone of the armies is under the direction of the Director-General of the Army Medical Service, who is attached to the War Office, and whose position corresponds to that of Surgeon-General in the U. S. Army. He controls the so-called reserve hospitals, which are scattered all over the kingdom. Aside from these there are also the territorial hospitals (about 160 in all with a total of 30,000 beds) which are under the direction of the Italian Red Cross. There were at the beginning of spring, 1918, 330,000 beds in Italy, which, added to the 700,000 beds existing within the zone of the armies, make the total military hospital capacity of Italy more than a million beds.

Under the Director-General of the Army Medical Service are 12 colonels of the Medical Corps, each attached to an Army Corps and with jurisdiction over the hospitals within this Army Corps. The 1250 hospitals have been distributed among the Army Corps according to the distance of the latter from the battle front, the building and transportation facilities, and the climate. In each A. C. district there is a hospital named " principale " with which all the others are connected. Most of the hospitals are for general patients but a number are reserved for certain classes of patients. The number of categories and branches which are successfully operating is impressive.

The Neuropsychiatric Service is one of the best of the Italian Army and consists of the following branches :

*The Neuropsychiatric Units in the Zones of the Armies.* These are mixed formations composed of :

1. Advanced Neuropsychiatric Sections.

2. Neuropsychiatric Centers of the Armies.

*The Neuropsychiatric Service Outside the War Zone* includes reserve military hospitals, university clinics, asylums, and constitutes :

1. The Psychiatric Service, dealing with *a)* Psychopathia requiring further clinical observation, or treatment, or medicolegal decision. *b)* Psychopathia having to be interned in asylums or state hospitals.

2. The Neurologic Centers. The largest centers are modeled on the French system and situated in : Pavia, Milan, Genoa, Bologna, Siena, Ancona, Rome, Naples, Bari and Catania.

About 20,000 patients are admitted per year to the psychiatric and neurologic reserve hospitals. According to Italian reports, 50 o/o of the patients of the 2nd Army were fit for duty after a short period of treatment and leave. Of the remaining 50 o/o, one-third had to be sent to psychopathic hospitals, but only one-half of these were declared insane and ultimately transferred to an asylum.

